# Involvement of *Lgals3*/Galectin-3 in Choroidal Neovascularization and Subretinal Fibrosis Formation

**DOI:** 10.3390/biomedicines12112649

**Published:** 2024-11-20

**Authors:** Di Wu, Ye Liu, Xiaogang Luo, Zhiqing Chen, Qiuli Fu, Ke Yao

**Affiliations:** Eye Center, The Second Affiliated Hospital, School of Medicine, Zhejiang University, Zhejiang Provincial Key Laboratory of Ophthalmology, Zhejiang Provincial Clinical Research Center for Eye Diseases, Zhejiang Provincial Engineering Institute on Eye Diseases, Hangzhou 310051, China; wudi0710@zju.edu.cn (D.W.); doctorliuye@zju.edu.cn (Y.L.); luoxiaogang@zju.edu.cn (X.L.); chenzhiqing@zju.edu.cn (Z.C.); 2313009@zju.edu.cn (Q.F.)

**Keywords:** Lgals3/galectin-3, age-related macular degeneration, choroidal neovascularization, subretinal fibrosis, hypoxia-inducible factor

## Abstract

**Background:** *Lgals3*/galectin-3 plays a pivotal role in many vascular diseases. However, the involvement of *Lgals3*/galectin-3 in eyes with neovascular age-related macular degeneration (nAMD) remains unknown. **Methods:** In the laser-induced CNV model, a whole mount retina stained with Isolectin B4 and collagen type I revealed the vascular bed and CNV-associated subretinal fibrosis on day 7 after laser treatment. **Results:** We show that the expression levels of *Lgals3*/galectin-3 were significantly increased in the RPE/choroidal complex of CNV mice. An intravitreal injection of *Lgals3*-siRNA significantly suppressed the area of CNV and subretinal fibrosis, together with *Mcp-1* decline. The mixture of *Lgals3*-siRNA and Ranibizumab showed more efficiency than each drug used separately. Hypoxia induced *Lgals3*/galectin-3 production in ARPE-19 cells, which was reduced by the silencing hypoxia-inducible factor -1α (*Hif-1a*). **Conclusions:** Our data indicated that *Lgals3*/galectin-3 is involved in the pathogenesis of CNV and subretinal fibrosis, and *Lgals3*/galectin-3 could be a potential therapeutic target for nAMD.

## 1. Introduction

Age-related macular degeneration (AMD) is the leading cause of irreversible blindness worldwide in the elderly, which affects over 196 million people, with this number rising to 288 million by the year 2040 with the growth of the elderly population [1]. AMD is characterized by the progressive degeneration/dysregulation of the neuronal retina, choriocapillaris, and retinal pigment epithelium (RPE) complex. Clinically, AMD is classified into the neovascular form (wet AMD) and non-neovascular form (dry AMD). Wet AMD (also known as nAMD) is primarily characterized by the formation of choroidal neovascularization (CNV) in the macular region, ultimately resulting in the formation of a fibrotic scar [2,3]. Despite anti-vascular endothelial growth factor (VEGF) therapy as the first-line treatment in nAMD being able to stabilize or even enhance visual function, a large percentage of patients suffer from an incomplete response to anti-VEGF therapy, manifested as persistent subretinal or sub-retinal pigment epithelium (RPE) fluid; persistent or new hemorrhage; and/or progressive lesion fibrosis [4]. Thus, it is urgent to explore the mechanisms beyond VEGF-mediated choroidal neovascularization (CNV) and subretinal fibrosis in nAMD.

Etiologically, a decreased flow of oxygen from the chorio-capillaries causes hypoxia in the RPE cells, which accelerates degeneration. The expression of HIFs (hypoxia-inducible transcription factors) has been detected in CNV membranes from nAMD patients [5]. The principal explanations for the expression of HIF in human CNV are that drusen maculopathy and the age-related thickening of the Bruch’s membrane (BM) together contribute to accelerated RPE hypoxia, and therefore cause increased levels of HIF transcription factors [6]. Moreover, a large number of hypoxia-induced factor (HIF) target genes contribute to nAMD, such as angiopoietins and plasminogen activator inhibitor 1 (PAI-1) [7,8]. In addition, hypoxia causes an inflammatory reaction in RPE cells, which are closely linked to angiogenesis and fibrosis. During this process, the inflammatory mediators such as monocyte chemoattractant protein-1/chemoattractant CC chemokine ligand 2 (MCP-1/CCL-2) were important in the inflammation reaction of nAMD [9,10].

Recently, we reported that *Lgals1*/galectin-1, as the first member of the galectin family, plays a key role in both angiogenesis and subretinal fibrosis in eyes with nAMD [11]. Galectins, which are carbohydrate-binding proteins, were discovered for their ability to bind galactoside in order to identify proteins that can decode complex cell-surface glycans [12]. Currently, 15 members of the galectin family are found in mammals, and they are widely distributed in different types of cells and tissues [13]. They are characterized as a protein family due to the conserved β-galactoside-binding sites found within their unique ~130 amino acid (aa) carbohydrate recognition domains (CRDs) and their participation in cell activation, differentiation, proliferation, and apoptosis. Different from other subtypes, *Lgals3*/galectin-3, as the only chimera-type galectin, was involved in various physiological functions, such as proliferation, apoptosis, differentiation, cellular adhesion, and tissue repair. Due to its multifunctional properties, *Lgals3*/galectin-3 plays different roles in several diseases, such as cancer, chronic inflammation, atherosclerosis, and cardiovascular diseases [14,15,16]. For instance, *Lgals3*/galectin-3 promotes EMT in a carbohydrate-dependent manner and enhances the metastatic potential of cancer cells [17]. In the eye, *Lgals3*/galectin-3 has been observed in migrating corneal epithelial cells [18], inflammatory conjunctival epithelium [19], and differentiated RPE cells [20]. So far, in vivo and in vitro studies indicated that *Lgals3*/galectin-3 plays critical roles in several ocular diseases, e.g., dry eye, glaucoma, uveitis, and diabetic retinopathy [21,22,23]. Recently, *Lgals3*/galectin-3 was found to be highly expressed in RPE cells from AMD donors than in age-matched healthy donors [24]. However, little is known about the alteration of *Lgals3*/galectin-3 expression in the retina in nAMD. In this study, we investigated the involvement of *Lgals3*/galectin-3 in CNV and subretinal fibrosis using a CNV mouse model and RPE cell culture.

## 2. Materials and Methods

### 2.1. Animals

In this study, C57BL/6 mice (6–8 weeks old) were supplied by the Laboratory Animal Center of Hangzhou Medical College (Hangzhou, China), and fed with standard laboratory food and water in a condition with a 12 h light-dark cycle. All animal experiments were conducted in accordance with the Association for Research in Vision and Ophthalmology’s Statement for the Use of Animal in Ophthalmic and Vision Research and were approved by the Institutional Animal Care and Use Committee at Zhejiang University.

### 2.2. Laser-Induced CNV Model

Mice were anesthetized with 1% pentobarbital, and their pupils were dilated with 0.5% tropicamide and 0.5% phenylephrine hydrochloride eyedrops (Santen Pharmaceutical Co., Osaka, Japan). Two minutes after pupil dilation, laser photocoagulation (532 nm, 400 mW, 80 ms) was induced in four spots per eye around the optic disk using the image-guided laser system (Micron IV, Phoenix Reserch Laboratories, Pleasanton, CA, USA). Immediately after laser injury, intravitreal injections were performed as described previously [25]. In brief, 1 μL of *Lgals3*-siRNA (Genechem, Shanghai, China) was diluted to 100 pM and intravitreally injected into right eye (1 μL/eye). In control group, 1 μL negative control siRNA (Con-siRNA) or/and Ranibizumab (Lucentis, Genentech, Inc., South San Francisco, CA, USA) was intravitreally injected into left eye.

### 2.3. Fundus Fluorescein Angiography and Optical Coherence Tomography

Procedures were described in previous report [26]. Fundus fluorescein angiography (FFA) was used to determine leakage by the retinal imaging microscope (Micron IV) 7 days after laser photocoagulation. In brief, mice were anesthetized, pupils dilated, and then intraperitoneally injected with sodium fluorescein (Sigma-Aldrich, Vienna, Austria). Fluorescent fundus images were captured using the retinal imaging microscope 5 min after fluorescein injection. Spectral domain optical coherence tomography (SD-OCT) was performed with the guidance of bright-field live fundus image using the image-guided OCT system (Micron IV). The process was performed following FFA detection and utilizing the Vendor’s image acquisition software (Discover 2.4.11) to capture bright-field images.

### 2.4. Assessment of CNV and Subretinal Fibrosis

Procedures were described in our previous study [11]. Seven days after laser photocoagulation, the eyes were removed and fixed in 4% paraformaldehyde. The anterior segment and the retina were removed from the eyeball, and then RPE/choroid complex was fluorescently labeled with markers for endothelial cells (Isolectin B4-Alexa 488, 1:100, Thermo Fisher Scientific, Tewksbury, MA, USA) and fibroblasts (rabbit anti-collagen type I antibody, 1:100, Abcam, Cambridge, MA, USA) to detect CNV and subretinal fibrosis, respectively. The images were obtained by fluorescence microscope (Leica, Wetzlar, Germany), and size of CNV and subretinal fibrosis were calculated by measuring the fluorescent areas using ImageJ Version 1.54 [National Institutes of Health (NIH), Bethesda, MD, USA].

### 2.5. Validation of Results

Selected genes were further validated seven days after laser injury through Quantitative real-time PCR (qRT-PCR). Following the manufacturers’ instructions, total RNA was isolated using TRIzol Reagent. Reverse transcription was then performed for cDNA using PrimeScript RT Master Mix (Takara, Beijing, China). Real-time qPCR was carried out using the SYBR Premix Ex Taq (Takara) with the ABI Fast 7500 RT-PCR system (Life Technologies, Grand Island, NY, USA). Gene expression levels were calculated using the 2-ddCT method, and gene levels were normalized using *Gapdh* as the internal control. Table 1 lists all primers.

### 2.6. Western Blot Analyses

Procedures were described in our previous study [25]. Protein extracts were obtained from two homogenized eyecups from each group seven days after laser treatment. Each sample was assessed for protein concentrations using Pierce BCA Protein Assay Kit (Thermo Fisher Scientific). Electrophoresis of proteins was performed using 12% SDS-polyacrylamide gels, with 20 μg of protein loaded on each lane. After the protein was electrotransferred to a 0.22 μm PVDF membrane (Millipore, Burlington, MA, USA), the membrane was blocked in Tris-buffered saline containing 5% skim milk and 0.1% Tween-20 and probed overnight at 4 °C with mouse anti-Galectin-3 antibody (Abcam, Fremont, CA, USA). Horseradish peroxidase-conjugated anti-mouse IgG (Thermo Fisher Scientific) was used as secondary antibodies for 1 h at room temperature. Immunoreactivity was visualized using enhanced chemiluminescence (ECL, Millipore) reagent. Protein levels were quantitated by densitometry and normalized to the β-actin levels.

### 2.7. Cell Culture

The human RPE cell line ARPE-19 was obtained from American Type Culture Collection (Manassas, VA, USA) and cultured in DMEM/F-12 medium from Gibco (Thermo Fisher Scientific, MA, USA), supplemented with 10% fetal bovine serum (FBS; Thermo Fisher Scientific). The following experiments were performed at 37 °C and 5% CO_2_, with either normoxia (20% O_2_) or hypoxia (1% O_2_ balanced with N_2_) in a humidified atmosphere. ARPE-19 cells were pretreated with 10nM LW6 (MedChemExpress, Monmouth Junction, NJ, USA) and placed in a hypoxic incubator for 24 h. DMSO was used as a negative control.

### 2.8. RNA-Seq Analysis

RNA of each sample was extracted from the RPE/choroidal complex using TRlzol Reagent (Life Technologies), as described in our previous study [27]. The purity and concentration of the total RNA samples were measured using NanoDrop 2000 Spectrophotometer (Thermo Fisher Scientific). RNA integrity was assessed using the RNA Nano 6000 Assay Kit on the Agilent Bioanalyzer 2100 system (Agilent Technologies, Santa Clara, CA, USA).

High-quality RNA samples were then sent to Biomarker Technologies Corporation (Hangzhou Cosmos Wisdom Biotechnology, Hangzhou, China) for the construction of cDNA libraries and sequencing. Following the manufacturer’s instructions, the RNA-seq libraries were prepared using the NEBNext UltraTM RNA Library Prep Kit for Illumina (New England Biolabs, Ipswich, MA, USA). Differential expression analysis of the two groups was conducted using DESeq2.

### 2.9. Statistical Analysis

All results were expressed as the mean ± standard error of the mean from at least three biological replicates. Statistical analyses were made by Student’s *t*-test or one-way ANOVA analysis. A value of *p* < 0.05 was considered statistically significant.

## 3. Results

### 3.1. Laser Irradiation Successfully Induced CNV and Subretinal Fibrosis

**Figure 1:**
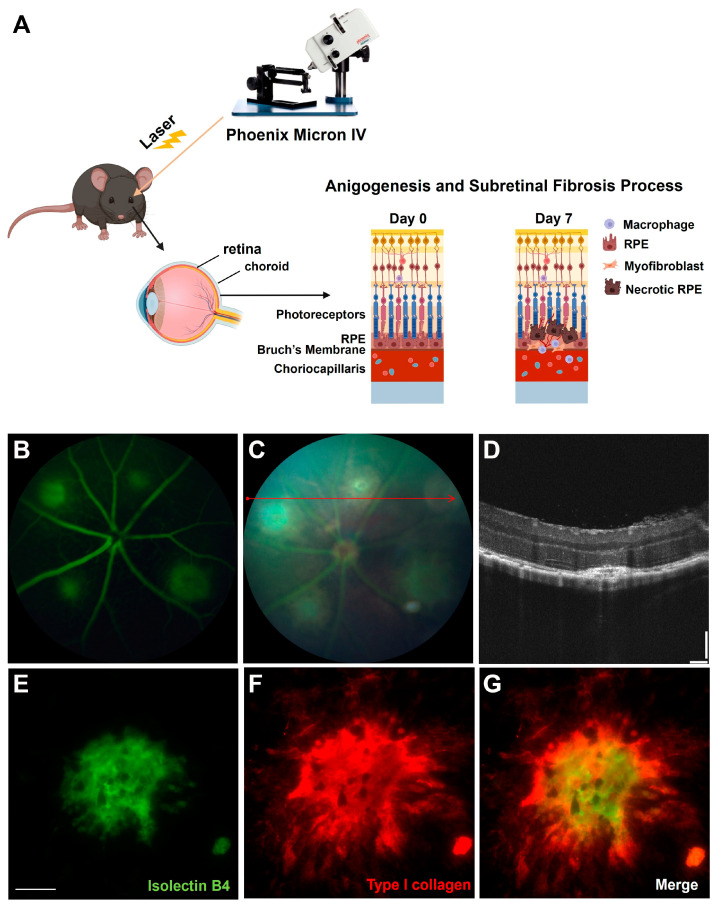
RNA-seq analysis of RPE/choroidal complex in laser injury mouse model at postlaser day 7. (**A**) A schema illustrating laser-induced CNV model in mice. (**B**) Fluorescein angiography of CNV model. (**C**) Fundus images of CNV model with cross-section OCT scans trace (red arrow). (**D**) Cross-sectional OCT scans of the lesion showing the disruption of BM at day 7. (**E**) CNV (Isolectin B4, green) image in laser-induced mouse model. (**F**) Subretinal fibrosis (Type I collagen, red) image in laser-induced mouse model. (**G**) Merge image of CNV and subretinal fibrosis lesions in laser-induced mouse model. Scale bar, 100 μm.

The laser-induced CNV mouse model demonstrates choroidal angiogenesis and subretinal fibrosis under burn-induced inflammation, mimicking some aspects of nAMD [27]. To confirm the feasibility of the laser-induced CNV model, the mouse fundus was viewed using an imaging camera, and laser photocoagulation burned Bruch’s membrane with an image-guided laser system (Schematic illustration of Laser-induced CNV mouse model) (Figure 1A). We first performed the FFA and OCT to show the vascular leakage and newly formed subretinal CNV at 7 days after laser injury (Figure 1B–D). Consistent with these findings, the Isolectin B4 and Type I collagen staining represented neovascular and fibrous tissue, respectively, which were identified to be surrounding laser spots in mice (Figure 1E–G).

### 3.2. Increase in Galectin-3 from RPE Cells in Laser-Induced CNV Mice

**Figure 2:**
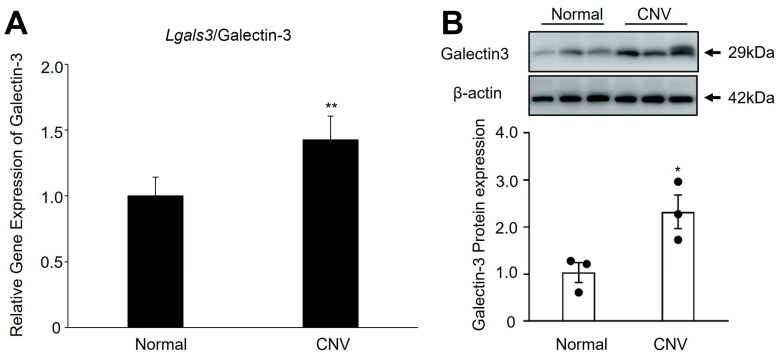
Upregulation of Lgals3/galectin-3 in RPE/choroidal complex of CNV model. (**A**) Lgals3 mRNA expression in RPE/choroidal complex of CNV and non-treated mice. (**B**) Western blot analysis of Galectin-3 in the RPE/choroidal complex of mice with CNV at postlaser day 7. n = 3 in each group. ** *p* < 0.01, * *p* < 0.05.

To determine the involvement of Lgals3/galectin-3 in CNV formation, real-time PCR and immunoblot were performed to determine the expression patterns of Lgals3/galectin-3 in the RPE/choroid complex of mice with laser-induced CNV. As shown in Figure 2, the gene and protein expression level of Lgals3/galectin-3 was significantly upregulated (gene, 1.42-fold change; protein, 2.47-fold change) at postlaser day 7 (Figure 2A,B). Taken together, our data suggested that Lgals3/galectin-3 might be involved in CNV and subretinal fibrosis formation in nAMD.

### 3.3. Attenuation of CNV and Subretinal Fibrosis Formation by Silencing of Galectin-3

**Figure 3:**
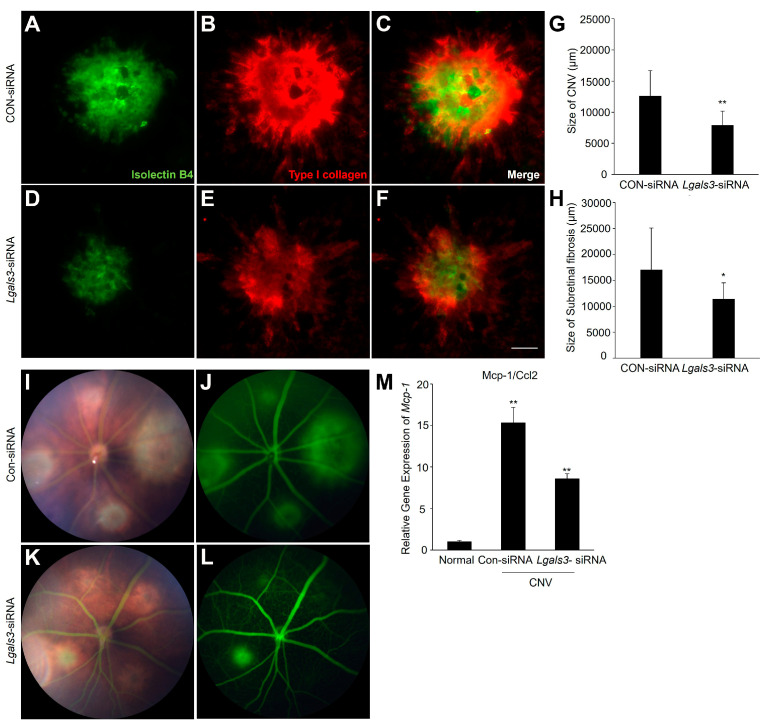
Silencing of Lgals3 suppressed the area of CNV and subretinal fibrosis, together with Mcp-1 and Icam-1 levels decline. (**A**–**F**) Representative images of CNV (Isolectin B4, green) and subretinal fibrosis (Type I collagen, red) in RPE/choroidal complex of CNV mice with or without Lgals3-siRNA intravitreal injection. Scale bar, 100 μm. (**G**,**H**) Quantitative analyses for the area of CNV [CON-siRNA = 12,671 ± 3392, Lgals3-siRNA = 17,110 ± 7979] and subretinal fibrosis [CON-siRNA = 17,110 ± 7979, Lgals3-siRNA = 11,481 ± 3053]. (**I**–**L**) Fundus images and fluorescein angiography of CNV model with Con-siRNA or Lgals3-siRNA. (**M**) The mRNA expression levels of Mcp-1 in RPE/choroidal complex of CNV mice with or without Lgals3-siRNA intravitreal injection. n = 5 in each group. * *p* < 0.05, ** *p* < 0.01.

To investigate the impact of *Lgals3*/galectin-3 in the development of CNV and subretinal fibrosis, we first examined the gene silencing efficiency of *Lgals3*-siRNA in ARPE-19 cells (Supplementary Figure S1). We then measured the size of CNV and subretinal fibrosis, with or without an *Lgals3*-siRNA intravitreal injection after laser injury. Isolectin-B4 and Type I collagen staining of choroidal flat mounts showed that the CNV and subretinal fibrosis areas of *Lgals3*-siRNA injection were decreased by approximately 37% and 33%, respectively, compared with that of Con-siRNA injection at 7 days after laser photocoagulation (Figure 3A–H). Meanwhile, pathological leakage resembling CNV formation occurred in most of the Con-siRNA injection mice, but rarely in the *Lgals3*-siRNA group (Figure 3I–L).

The laser-induced CNV model is similar to a wound-healing reaction, accompanied with high levels of acute reaction inflammation [28]. To explore whether the inhibition of *Lgals3* suppresses angiogenesis and fibrosis via an inflammatory pathway, we measured the changes in *Mcp-1*/*Ccl-2* in CNV mice with or without *Lgals3*-siRNA injection. As shown in Figure 3M, the silencing of *Lgals3* exhibited a significant reduction in *Mcp-1*/*Ccl-2* in the RPE/choroidal complex at postlaser day 7. These data suggested that *Lgals3*/galectin-3 promotes CNV and subretinal fibrosis development, at least partially via the inflammatory signaling pathway.

### 3.4. Comparing the Inhibitory Effects of Lgals3/Galectin-3 and Lucentis on CNV and the Formation of Subretinal Fibrosis

**Figure 4:**
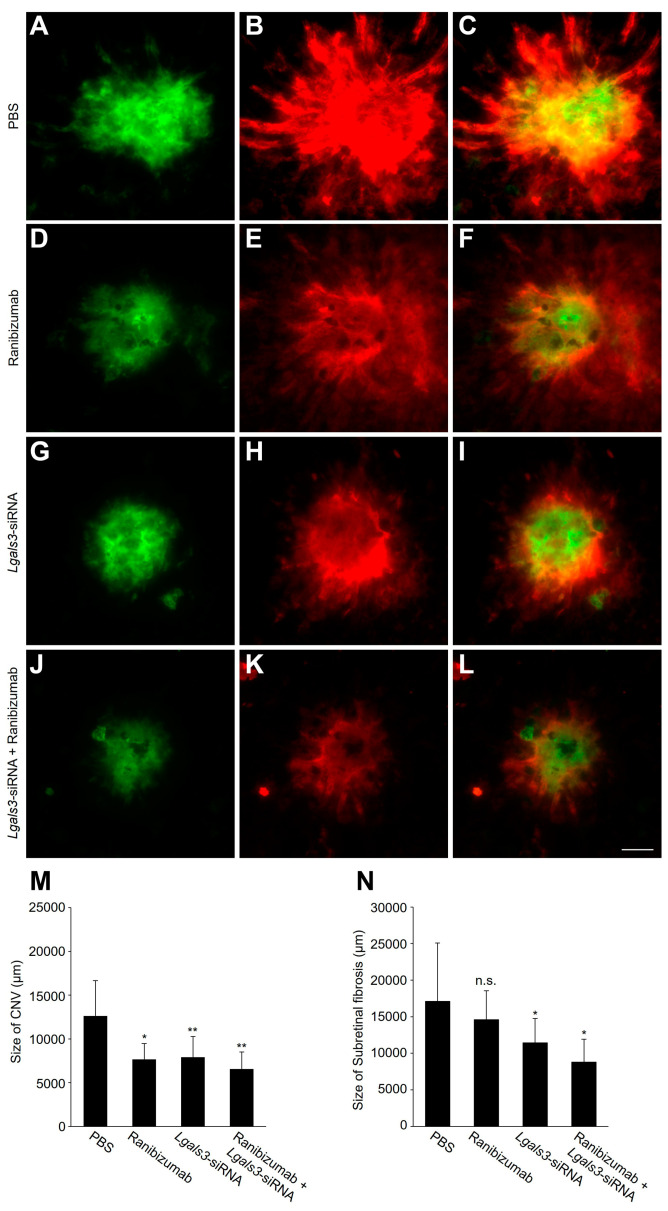
Comparison of Lgals3-siRNA and Ranibizumab for suppression of CNV and subretinal fibrosis development. (**A**–**L**) Representative micrographs of CNV (Isolectin B4, green) and subretinal fibrosis (Type I collagen, red) in the RPE/choroidal complex of CNV mice treated with intravitreal injection of PBS (**A**–**C**), Ranibizumab (**D**–**F**), Lgals3-siRNA (**G**–**I**), and mixture of Ranibizumab + Lgals3-siRNA (**J**–**L**), respectively. Scale bar, 100 μm. n = 6. (**M**,**N**) Quantification analysis of the sizes of CNV [PBS =12,671 ± 3992, Ranibizumab = 7697 ± 1785, Lgals3-siRNA = 7959 ± 2308, Lgals3-siRNA + Ranibizumab = 6616 ± 1903] and subretinal fibrosis [PBS =17,110 ± 7979, Ranibizumab = 14,583 ± 3983, Lgals3-siRNA =11,422 ± 3333, Lgals3-siRNA + Ranibizumab = 8797 ± 3142]. * *p* < 0.05, ** *p* < 0.01. n = 6. n.s., not significant.

Since *Lgals3*/galectin-3 was shown as a possible molecular target for nAMD, we then compared the efficiency of *Lgals3*/galectin-3 inhibition with that of Ranibizumab, one of the first-line drugs used to treat nAMD targeting VEGF [29]. In CNV mice, an intravitreal injection of PBS, Ranibizumab, *Lgals3*-siRNA and a combination of Ranibizumab and *Lgals3*-siRNA were used to quantify the size of CNV and subretinal fibrosis. As shown in Figure 4A–N, Ranibizumab treatment substantially reduced the size of CNV and subretinal fibrosis in mice, despite the reduction in subretinal fibrosis being without statistical significance. Moreover, a more remarkable inhibition was exhibited when the mice were treated with a mixture of *Lgals3*-siRNA and Ranibizumab compared to each drug being used separately.

### 3.5. Hypoxia Upregulates Galectin-3 Expression in RPE Cells via Hif-1a

**Figure 5:**
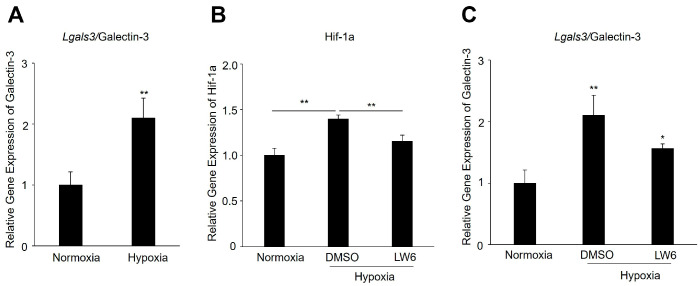
Expression of *Lgals3*/galectin-3 in ARPE-19 cells with silencing of *Hif-1a* under hypoxic conditions. (**A**) *Lgals3* mRNA expression at 24 h after hypoxic stimulation. (**B**) *Hif-1a* mRNA expression with *Hif-1a* inhibitor under hypoxic conditions. (**C**) *Lgals3* mRNA expression with or without *Hif-1a* inhibitor under hypoxic conditions. n = 5 in each group. * *p* < 0.05, ** *p* < 0.01.

To elucidate the molecular mechanism underlying *Lgals3*/galectin-3 upregulation in CNV, we conducted in vitro experiments utilizing human RPE cells subjected to hypoxic conditions, which are used to mimic an in vivo study of nAMD, to examine the changes in *Lgals3* gene expression under hypoxic conditions. As shown in Figure 5A, the mRNA expression level of *Lgals3* significantly increased to 2.1 times (*p* < 0.01) with hypoxic stimulation.

HIF is the master regulator factor mediated by molecular oxygen [30]. Since the oxygen level is low, HIF-1α regulates the expression of various critical downstream genes that are necessary for cells to survive. To understand whether Hif-1a plays a role in controlling *Lgals3* expression under hypoxic conditions, we analyzed the mRNA expression levels of *Lgals3* while using a Hif-1a inhibitor. As demonstrated in Figure 5B, LW6, a suppressor of Hif-1a accumulation, significantly suppressed the mRNA expression levels of *Hif-1a* under hypoxic conditions. Likewise, the gene expression levels of *Lgals3* were greatly reduced by LW6 (Figure 5C). These results indicate that hypoxia influences the transcriptional activity of *Lgals3*, showing its dependence on the regulation by *Hif-1a.*

### 3.6. Bioinformatics Analysis of Differentially Expressed Genes and Pathways in RPE Cells by CNV Induction

**Figure 6:**
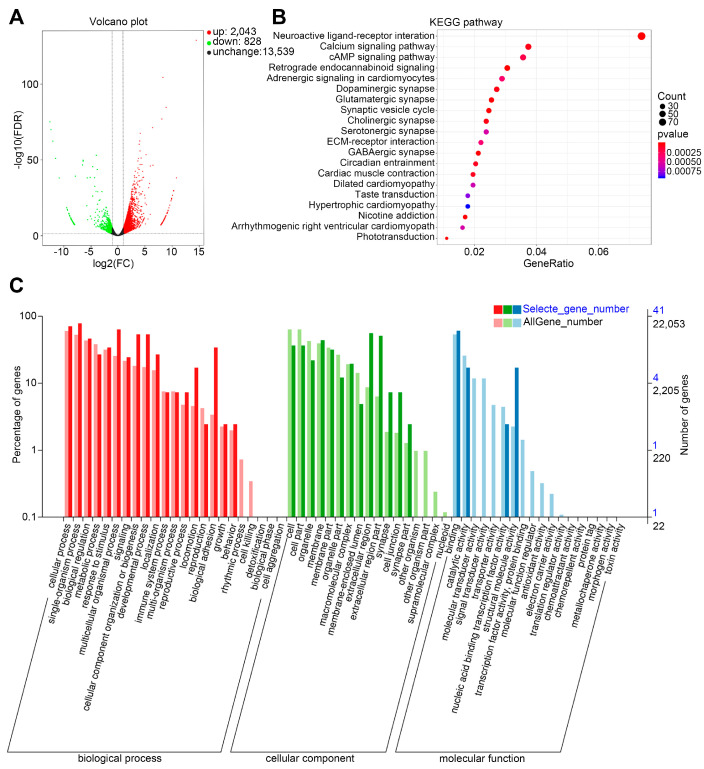
Identification of DEGs and pathways in RPE cells by CNV induction. (**A**) The volcano plot of differentially expressed genes in RPE/choroidal complex of CNV mode. (**B**) The GO enrichment analysis bar diagram. (**C**) The KEGG enrichment analysis scatter plot.

RNA-seq is the technique that uses the capabilities of high-throughput sequencing to reveal the presence and quantity of RNA molecules in a biological sample and look at alternative genes in different groups or treatments [31]. We finally performed RNA-seq analysis to investigate changes in the gene expression profiles of the choroidal CNV sample. In total, 2871 significant differentially expressed genes (DEGs) were identified in the RPE/choroidal complex between laser-induced CNV and control mice, which consisted of 2043 upregulated and 828 downregulated genes (Figure 6A) (details in Supplementary Excel S1). We observed that *Hif-1* and *Lgals3*/Galectin-3 were increased in laser-induced CNV mice (fold change with CNV: *Hif-1* = 1.50, *Lgals3*/Galectin-3 = 1.78). Moreover, the upregulated genes, such as Muc4, Septin4, Alox15, Dsg3, and Ska2, encoded pro-inflammatory and apoptotic mediators, and cytoskeleton components were confirmed through this experimental validation. Then, the GO and KEGG enrichment analysis were obtained based on DEGs and indicated that the Neuroactive ligand-receptor interaction pathway, Calcium signaling pathway, and cAMP signaling pathways were the primary pathways that were likely responsible for laser-induced CNV mice (Figure 6B,C).

## 4. Discussion

The current study reveals, for the first time, several important findings concerning the involvement of *Lgals3*/galectin-3 in the pathogenesis of CNV and subretinal fibrosis. Our initial process included establishing a stable laser-induced CNV mouse model (Figure 1). Our recent study provided evidence that *Lgals1*/galectin-1 could modify the VEGFA and TGF-beta1 receptor signaling pathways, thus altering nAMD [11]. This prompted us to investigate if other galectin family members are involved in nAMD. As members of the galectin family, galectin-1 and galectin-3 are ubiquitously expressed with various biological roles [32]. The difference with galectin-1 is that galectin-3 contains one CRD with an atypical N-terminal domain, which suggests functional differences between these two subtypes. Galectin-3 contains one CRD with an atypical N-terminal domain, which suggests functional differences between these two subtypes. Recently, *Lgals3*/galectin-3 has been reported to significantly interact with endothelial cells [33], RPE [34], microglial [22], and macrophages [35] in the retina. In this study, we indicated that *Lgals3*/galectin-3 resulted in an increase in mRNA and protein levels in the laser-induced CNV model (Figure 2), suggesting that *Lgals3*/galectin-3 might play a role in nAMD.

Neovascularization and subretinal fibrosis have been revealed as key pathological events associated with poor visual acuity in nAMD [2]. The previous study has shown that *Lgals3*/galectin-3 triggers FAK signaling pathways by binding to cell adhesion receptor αvβ3 integrin, thus promoting endothelial cell migration in the angiogenic cascade [36]. Additionally, *Lgals3*/galectin-3 has been seen to hinder the internalization of VEGFR in human umbilical vein endothelial cells when bound to VEGFR2, thereby stimulating pathological angiogenesis in the cornea [37]. In agreement with these findings, a recent study has shown that the silencing of *Lgals3*/galectin-3 could suppress corneal neovascularization and fibrosis through α-smooth muscle actin (α-SMA) signaling in a silver nitrate cautery and alkaline burn mouse model [38]. Importantly, *Lgals3*/galectin-3 has been discovered extensively in RPE cells from AMD donors compared to age-matched healthy donors, suggesting that *Lgals3*/galectin-3 plays a role in the pathogenesis of nAMD [24,39].

Inflammation plays a critical role in CNV and subretinal fibrosis, as observed in the laser injury mouse model [40,41]. The process involves the activation of macrophages, leading to an increase in cytokine and chemokine levels. Our prior research showed that macrophage influx into the RPE/choroidal complex and the expression level of *MCP-1/CCL2* significantly increased in mice with CNV [25]. It is known that persistent pro-inflammatory cytokines released from RPE cells and macrophages promote the differentiation and activation of myofibroblasts (e.g., EndMT and EMT) [2,11]. The progression culminates in subretinal scarring, causing irreversible destruction of photoreceptors, RPE cells, and choroidal blood vessels. Recently, *Lgals3*/galectin-3 has been shown to regulate the balance between pro-inflammatory and anti-inflammatory effects of microglia in neurodegenerative diseases [42]. Our current study aligns with previous findings, highlighting the effects of blocking *Lgals3*/galectin-3 on the progression of CNV and subretinal fibrosis, along with the downregulation of *Mcp-1/Ccl-2* (Figure 3). The current study suggested that *Lgals3*/galectin-3 could be a potential target gene to intensify inflammation-induced angiogenesis and subretinal fibrosis in the retina, which is driven in part by *MCP-1/CCL-2* signaling pathway. Moreover, the combination therapy with anti-VEGF and *Lgals3*-siRNA was shown to be more effective than each drug being used separately (Figure 4).

Finally, we explored the molecular mechanism behind the upregulation of *Lgals3*/galectin-3 in CNV. Research has indicated that the RPE, a central cellular actor in the development of nAMD, plays significant roles in angiogenesis and the formation of subretinal fibrotic scars [43]. It is known that hypoxia plays a central role in causing CNV formation as it triggers the activation of the HIF family, which subsequently stimulates a broad range of genes [44]. Recent work indicated that hypoxia promotes *Lgals3* activity depending on HIF-1α in renal carcinoma cells [45]. The promoter region of the human *Lgals3* gene contains several regulatory elements, such as AP-1, cAMP-dependent response element (CRE) motifs, and a consensus basic helix–loop–helix (bHLH) core sequence [46]. In our study, we identified that the promoter sequence of the *LGALS3* gene contained three hypoxia response elements (HREs) (Supplementary Data Figure S2), which might be involved in the regulation of *Lgals3*/galectin-3 by HIF-1α. Our in vitro study confirmed that LW6, a selective inhibitor of *Hif-1a*, significantly reduced the production of *Lgals3*. This suggests that an increase in *Lgals3*/galectin-3 could be stimulated by *Hif-1a* in RPE cells under hypoxic conditions (Figure 5). Furthermore, we performed an RNA-seq analysis on RPE/choroidal complex cells at postlaser day 7 and identified 2043 upregulated and 828 downregulated genes. These genes also regulate a number of biological pathways in the progression of CNV and subretinal fibrosis, such as the Neuroactive ligand-receptor interaction pathway, Calcium signaling pathway, and cAMP signaling pathways (Figure 6). These pathways need to be further studied.

**Figure 7:**
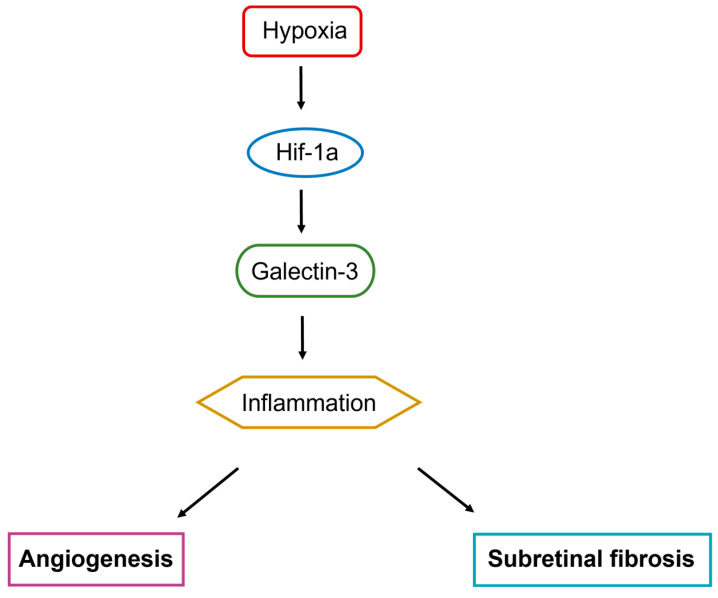
Galectin-3 involvement in the molecular pathogenesis of CNV.

In summary, in this study, we propose that HIF-1α binds HREs at the promoter region of *Lgals3*/galectin-3 to induce its expression under hypoxia in human RPE cells. Moreover, we highlight the role of *Lgals3*/galectin-3 mediated angiogenesis and subretinal fibrosis through inflammatory response in a laser-induced mouse model (Figure 7).

There are also some limitations to consider in this study. So far, several animal models have been demonstrated to mimic the pathological features commonly seen in AMD, including mouse models with oxidative damage and genetic mouse models. However, no one model recapitulates all of the features observed in AMD patients. All discoveries of *Lgals3*/galectin-3 were proved in the CNV mouse model, but not in other animal models. Additionally, Lgals3-siRNA has shown promising results for suppressing CNV and subretinal fibrosis; however, it would be difficult to deliver to the inner layer of the retina. Therefore, developing an effective drug delivery probably needs to be performed in future studies.

## 5. Conclusions

The development of angiogenesis and subretinal fibrosis in nAMD has become increasingly important since it significantly impacts the success of nAMD therapy. However, the molecular pathogenesis remains unclear. Our findings provide novel insights into the involvement of *Lgals3*/galectin-3 in nAMD. These findings indicated that *Lgals3*/galectin-3 plays an important role in the progression of nAMD, and it might be a promising therapeutic target of nAMD.

## Figures and Tables

**Table 1:** Primers used for real-time PCR amplification.

	Primer, 5′-3′	
Gene	Forward	Reverse
*Lgals3*	AACACGAAGCAGGACAATAACTGG	GCAGTAGGTGAGCATCGTTGAC
*Hif-1a*	TGCTCATCAGTTGCCACTTC	TGGGCCATTTCTGTGTGTAA
*Ccl2*	TTGGCTCAGCCAGATGCA	CCTACTCATTGGGATCATCTTGC
*Gapdh*	AGGTCGGTGTGAACGGATTTG	TGTAGACCATGTAGTTGAGGTCA

## Data Availability

The original contributions presented in the study are included in the article, further inquiries can be directed to the corresponding author.

## Supplementary Materials

The following supporting information can be downloaded at: https://www.mdpi.com/article/10.3390/biomedicines12112649/s1, Figure S1: Efficiency of Lgals3-siRNA in ARPE-19 cells; Figure S2: Analyzation of HRE sites in GALECTIN-3/*LGALS3* promoter. Excel S1: COV-vs-CON_DE_results.
