# Supplementary material for: Involvement of Lgals3/Galectin-3 in Choroidal Neovascularization and Subretinal Fibrosis Formation
Source: Biomedicines. 2024 Nov 20;12(11):2649. doi: 10.3390/biomedicines12112649 (PMC11592115; doi:10.3390/biomedicines12112649)
Supplement: Supplementary file 1 [file biomedicines-12-02649-s001.zip › Supplementary data/Supplementary data V1.pptx]

## Slide 1
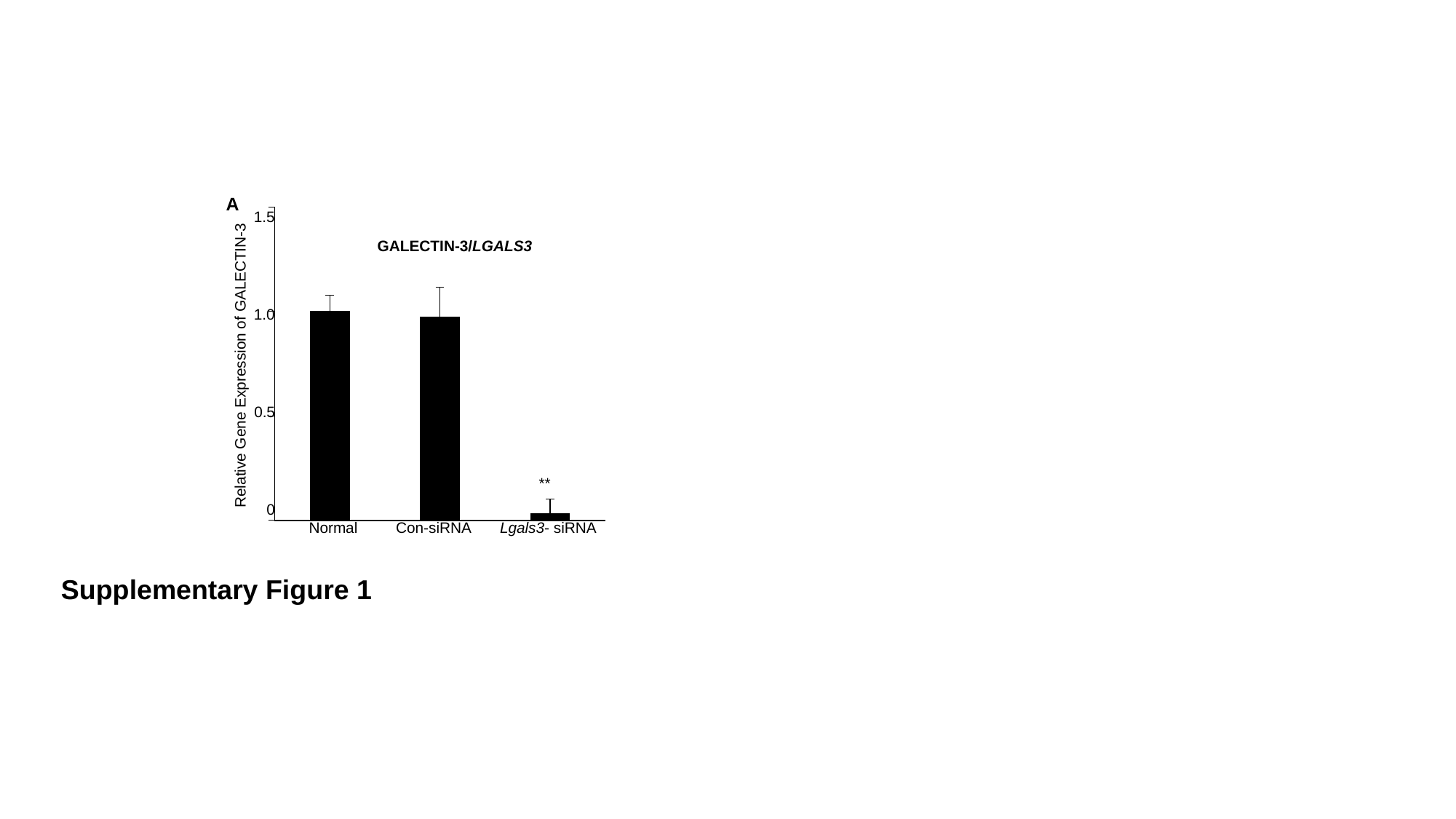

A
### Chart
| Category | |
|---|---|
| ARPE-19 | 1.0 |
| CON-siRNA | 0.9733846211734455 |
| Lgals-3 siRNA | 0.03175146263854254 |1.5
GALECTIN-3/LGALS3
1.0
Relative Gene Expression of GALECTIN-3
0.5
**
0
Con-siRNA
Lgals3- siRNA
Normal
Supplementary Figure 1

## Slide 2
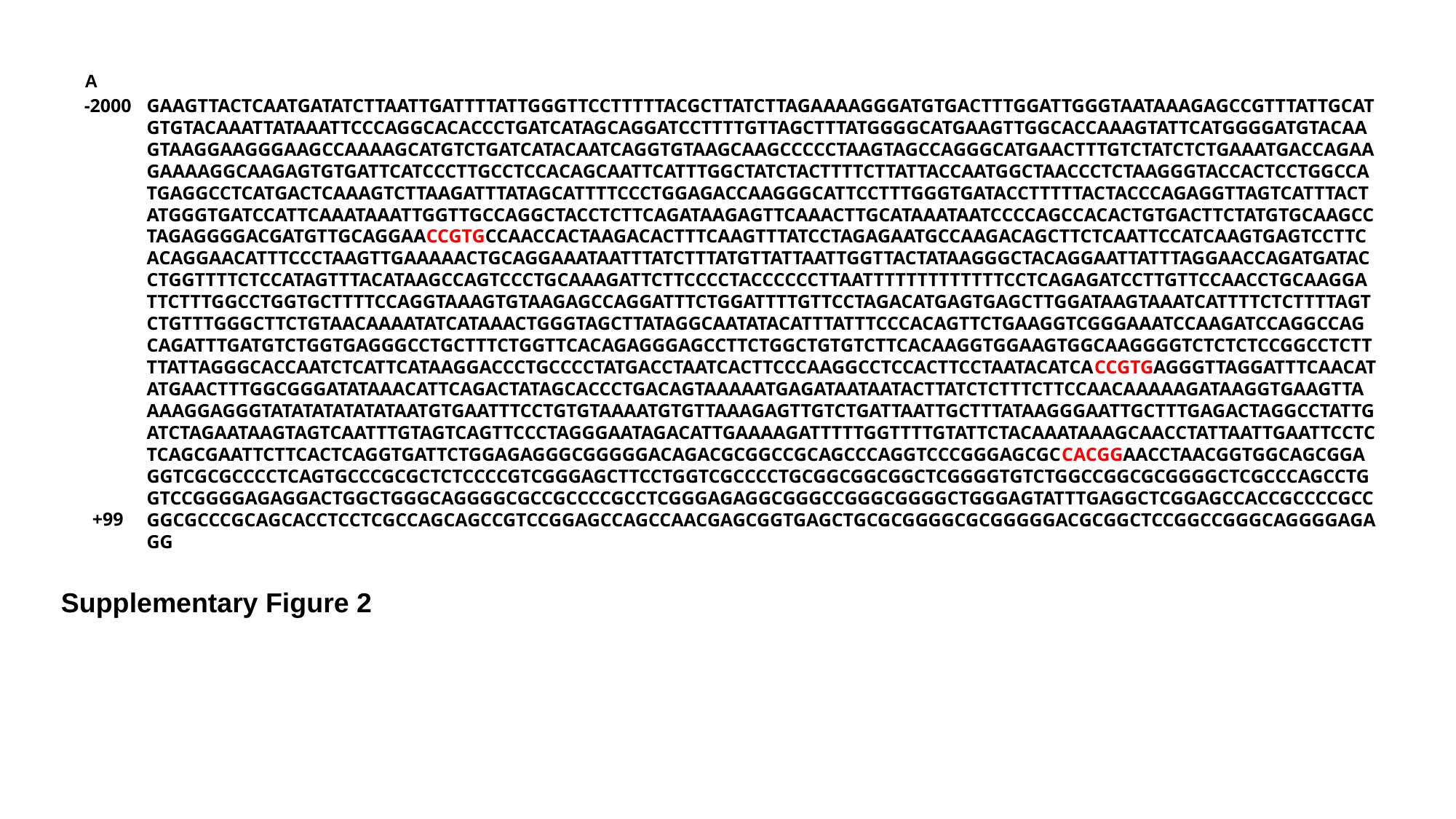

A
-2000
GAAGTTACTCAATGATATCTTAATTGATTTTATTGGGTTCCTTTTTACGCTTATCTTAGAAAAGGGATGTGACTTTGGATTGGGTAATAAAGAGCCGTTTATTGCATGTGTACAAATTATAAATTCCCAGGCACACCCTGATCATAGCAGGATCCTTTTGTTAGCTTTATGGGGCATGAAGTTGGCACCAAAGTATTCATGGGGATGTACAAGTAAGGAAGGGAAGCCAAAAGCATGTCTGATCATACAATCAGGTGTAAGCAAGCCCCCTAAGTAGCCAGGGCATGAACTTTGTCTATCTCTGAAATGACCAGAAGAAAAGGCAAGAGTGTGATTCATCCCTTGCCTCCACAGCAATTCATTTGGCTATCTACTTTTCTTATTACCAATGGCTAACCCTCTAAGGGTACCACTCCTGGCCATGAGGCCTCATGACTCAAAGTCTTAAGATTTATAGCATTTTCCCTGGAGACCAAGGGCATTCCTTTGGGTGATACCTTTTTACTACCCAGAGGTTAGTCATTTACTATGGGTGATCCATTCAAATAAATTGGTTGCCAGGCTACCTCTTCAGATAAGAGTTCAAACTTGCATAAATAATCCCCAGCCACACTGTGACTTCTATGTGCAAGCCTAGAGGGGACGATGTTGCAGGAACCGTGCCAACCACTAAGACACTTTCAAGTTTATCCTAGAGAATGCCAAGACAGCTTCTCAATTCCATCAAGTGAGTCCTTCACAGGAACATTTCCCTAAGTTGAAAAACTGCAGGAAATAATTTATCTTTATGTTATTAATTGGTTACTATAAGGGCTACAGGAATTATTTAGGAACCAGATGATACCTGGTTTTCTCCATAGTTTACATAAGCCAGTCCCTGCAAAGATTCTTCCCCTACCCCCCTTAATTTTTTTTTTTTTCCTCAGAGATCCTTGTTCCAACCTGCAAGGATTCTTTGGCCTGGTGCTTTTCCAGGTAAAGTGTAAGAGCCAGGATTTCTGGATTTTGTTCCTAGACATGAGTGAGCTTGGATAAGTAAATCATTTTCTCTTTTAGTCTGTTTGGGCTTCTGTAACAAAATATCATAAACTGGGTAGCTTATAGGCAATATACATTTATTTCCCACAGTTCTGAAGGTCGGGAAATCCAAGATCCAGGCCAGCAGATTTGATGTCTGGTGAGGGCCTGCTTTCTGGTTCACAGAGGGAGCCTTCTGGCTGTGTCTTCACAAGGTGGAAGTGGCAAGGGGTCTCTCTCCGGCCTCTTTTATTAGGGCACCAATCTCATTCATAAGGACCCTGCCCCTATGACCTAATCACTTCCCAAGGCCTCCACTTCCTAATACATCACCGTGAGGGTTAGGATTTCAACATATGAACTTTGGCGGGATATAAACATTCAGACTATAGCACCCTGACAGTAAAAATGAGATAATAATACTTATCTCTTTCTTCCAACAAAAAGATAAGGTGAAGTTAAAAGGAGGGTATATATATATATAATGTGAATTTCCTGTGTAAAATGTGTTAAAGAGTTGTCTGATTAATTGCTTTATAAGGGAATTGCTTTGAGACTAGGCCTATTGATCTAGAATAAGTAGTCAATTTGTAGTCAGTTCCCTAGGGAATAGACATTGAAAAGATTTTTGGTTTTGTATTCTACAAATAAAGCAACCTATTAATTGAATTCCTCTCAGCGAATTCTTCACTCAGGTGATTCTGGAGAGGGCGGGGGACAGACGCGGCCGCAGCCCAGGTCCCGGGAGCGCCACGGAACCTAACGGTGGCAGCGGAGGTCGCGCCCCTCAGTGCCCGCGCTCTCCCCGTCGGGAGCTTCCTGGTCGCCCCTGCGGCGGCGGCTCGGGGTGTCTGGCCGGCGCGGGGCTCGCCCAGCCTGGTCCGGGGAGAGGACTGGCTGGGCAGGGGCGCCGCCCCGCCTCGGGAGAGGCGGGCCGGGCGGGGCTGGGAGTATTTGAGGCTCGGAGCCACCGCCCCGCCGGCGCCCGCAGCACCTCCTCGCCAGCAGCCGTCCGGAGCCAGCCAACGAGCGGTGAGCTGCGCGGGGCGCGGGGGACGCGGCTCCGGCCGGGCAGGGGAGAGG
+99
Supplementary Figure 2
